# Panamanian Geisha Coffee Exhibits Antioxidant and Vasorelaxant Activities with a Favorable Safety Profile

**DOI:** 10.3390/foods15122172

**Published:** 2026-06-16

**Authors:** Kilmara Ábrego-González, Abdy Morales, Hugo A. Sánchez-Martínez, Maricselis Díaz, Aracelly Vega, Juan A. Morán-Pinzón, Jose Luis López-Pérez, Esther del Olmo, Estela Guerrero De León

**Affiliations:** 1 Universidad Autónoma de Chiriquí, David 0427, Panama; kilmara.abrego@up.ac.pa; 2 Centro de Investigaciones Psicofarmacológicas, Universidad de Panamá, Panama City 3366, Panama; abdy.morales@up.ac.pa (A.M.); hugo.sanchez02@up.ac.pa (H.A.S.-M.); maricselis.diaz@up.ac.pa (M.D.); juan.moran@up.ac.pa (J.A.M.-P.); 3 Departamento de Farmacología, Facultad de Medicina, Universidad de Panamá, Panama City 3366, Panama; lopez@usal.es; 4 Centro de Investigación en Recursos Naturales, Universidad Autónoma de Chiriquí, David 0427, Panama; aracelly.vega@unachi.ac.pa; 5 Sistema Nacional de Investigación de Panamá (SNI-SENACYT), Panama City 07144, Panama; 6 Departamento de Ciencias Farmacéuticas, Facultad de Farmacia, Universidad de Salamanca, 37007 Salamanca, Spain

**Keywords:** Panamanian Geisha coffee, phytochemical profile, antioxidant activity, vascular bioactivity, chlorogenic acids, functional beverage

## Abstract

Geisha coffee (*Coffea arabica* L. cv. Geisha) is internationally recognized for its exceptional sensory quality; however, its functional properties and bioactive composition remain insufficiently explored. This study evaluated the phytochemical profile, antioxidant capacity, vascular bioactivity, and toxicological safety of an aqueous extract of roasted Geisha coffee (AErGC) from the Chiriquí highlands, Panama. The chemical composition was determined using HPLC-PDA. Antioxidant activity was assessed using DPPH, ABTS, and lipid peroxidation assays. Vascular effects were studied in rat aortic rings, and safety was evaluated through *Artemia salina* and a single-dose acute oral toxicity model in rats (OECD 423). Chemical characterization was performed by HPLC-PDA, revealing notably elevated levels of caffeine (69.5 ± 6.4 mg/g) and 5-*O*-caffeoylquinic acid (74.5 ± 6.9 mg/g). The extract exhibited strong free radical scavenging capacity, with an IC_50_ value of 14.7 ± 4.9 µg/mL in the DPPH assay, and inhibited lipid peroxidation by 72.71 ± 1.63% at 15.6 µg/mL. In endothelium-intact rings, AErGC induced a concentration-dependent vasorelaxant effect, reaching a maximum relaxation of 70.84 ± 2.9%. Toxicological results showed an LC_50_ > 1000 µg/mL in *A. salina* and an oral LD_50_ > 2000 mg/kg, classifying the extract as Category 5 (low toxicity). These findings highlight Panamanian Geisha coffee as a promising functional beverage with antioxidant and vascular protective properties, supporting its potential as a nutraceutical.

## 1. Introduction

Driven by a shift toward precise cultivation and unique sensory profiles, the international coffee market is experiencing a significant evolution [1]. Within this context, specialty coffees have emerged as a premium segment, offering refined sensory experiences that appeal to highly discerning consumers [2]. Unlike commercial coffees, which prioritize high yields and uniformity, specialty coffees emphasize the unique characteristics associated with origin, varietal genetics, and post-harvest processing. To ensure consistent quality, the Specialty Coffee Association (SCA) has established internationally recognized standards, including strict limits on defects such as Quaker beans, which must be met for coffee to be marketed as “specialty” [2].

The production of premium coffee begins with the selection of cherries grown under optimal environmental conditions and harvested at peak maturity. Agroecological variables such as altitude, soil characteristics, and precipitation patterns significantly influence the biochemical composition of the beans, ultimately shaping their sensory properties [3,4,5,6]. Furthermore, post-harvest processing methods play a decisive role in defining attributes like acidity, body, and aromatic complexity [7,8]. Washed coffees are often associated with higher clarity and acidity, whereas natural processing often enhances body and complexity [9,10,11]. These sensory differences are attributed, in part, to the biochemical transformation of sugars and other compounds during processing [12]. The subsequent roasting process induces additional chemical reactions that profoundly shape both the flavor profile and bioactive composition of coffee [10].

Among commercially important species, *Coffea arabica* is the most widely consumed and has attracted growing interest due to its potential health benefits [13,14,15]. Of particular note is the Geisha variety; although native to Ethiopia, its cultivation in the Panamanian highlands has positioned it as a preeminent specialty coffee, consistently achieving record-breaking prices in international markets [16]. This economic and cultural impact is further underscored by its record-breaking auction prices, reaching USD 10,005 per kilogram in 2023 and rising dramatically to USD 30,204 per kilogram in 2025 [17]. Geisha coffee is distinguished by its exceptional sensory profile, characterized by floral, citrus, and bergamot notes that significantly differentiate it from other *Coffee arabica* varieties [18,19]. Recent studies have demonstrated that the unique organoleptic characteristics of Panamanian Geisha are influenced by both genetic factors and the specific terroir of production areas such as Boquete, Potrerillos Arriba, and Renacimiento, which bestows a distinctive regional identity upon this coffee [20]. Furthermore, beyond its sensory attributes, recent phytochemical investigations have revealed that both the leaves and green beans of the Geisha variety exhibit significantly high concentrations of bioactive compounds, including total phenols, flavonoids, and chlorogenic acid, surpassing other *Coffee arabica* cultivars in terms of antioxidant content [21].

Beyond its economic and sensory prestige, the Geisha variety possesses a rich secondary metabolite composition; particularly, its high concentration of phenolic compounds and terpenes, such as limonene, underscores its potential as a significant source of functional agents with antioxidant and anti-inflammatory activities [18,21]. Complementing these attributes, recent evidence highlights that the ratio of chlorogenic acids to caffeine (CGAs/Caf) may play a key role in mediating these health benefits [22]. A distinctive feature of Panamanian Geisha is its elevated CGAs/Caf ratio—ranging between 2.48 and 3.85—which further marks its potential for human health research and therapeutic applications [23].

Despite these promising bioactive attributes, a comprehensive pharmacological characterization of Panamanian Geisha coffee—particularly regarding its safety profile and vascular effects—remains limited. Consequently, the present study aimed to systematically characterize the phytochemical profile of an aqueous extract of roasted Panamanian Geisha coffee (AErPGC), as well as to evaluate its toxicity, antioxidant capacity, and vascular activity through complementary in vitro and in vivo experimental models. By establishing these safety and functional parameters, this research contributes to a deeper insight into the therapeutic potential of Geisha coffee, extending its value beyond its well-recognized sensory excellence.

## 2. Materials and Methods

### 2.1. Chemicals and Reagents

All chemicals, drugs, and reagents used in this investigation were of analytical grade. Phenylephrine hydrochloride (PE), acetylcholine chloride (ACh), dimethyl sulfoxide (DMSO), methanol, ethanol, ferric chloride (FeCl_3_), formic acid, acetonitrile (ACN), Folin–Ciocalteu reagent, sodium carbonate (Na_2_CO_3_), potassium ferricyanide (K_3_Fe(CN)_6_), Griess reagent, sodium nitroprusside (SNP), 2,2-diphenyl-1-picrylhydrazyl (DPPH), potassium persulfate (K_2_S_2_O_8_), 2,2-azino-bis(3-ethylbenzothiazoline-6-sulphonic acid) (ABTS), ascorbic acid (vitamin C), 6-hydroxy-2,5,7,8-tetramethylchroman-2-carboxylic acid (Trolox), acetic acid, caffeic acid, gallic acid, tannic acid, trichloroacetic acid (TCA), trifluoroacetic acid, vanillic acid, protocatechuic acid, quercetin, nitroblue tetrazolium (NBT), nicotinamide adenine dinucleotide (NADH), sodium nitrite (NaNO_2_), aluminum chloride (AlCl_3_), ferrous sulfate (FeSO_4_), sodium hydroxide (NaOH), phenazine methosulfate (PMS), sodium chloride (NaCl), potassium chloride (KCl), monosodium phosphate (NaH_2_PO_4_), sodium bicarbonate (NaHCO_3_), calcium chloride (CaCl_2_), magnesium sulfate (MgSO_4_) and glucose were purchased from Sigma Chemical (Saint Louis, MO, USA). Artemia salina eggs were purchased from Benchmark Company and hatched using Instant Ocean Sea Salt from Spectrum Brands, Inc., Middleton, MI, USA.

### 2.2. Plant Material and Sample Preparation

Ripe cherries of *Coffea arabica* L. cv. Geisha were harvested between December 2021 and February 2022 from Jurutungo Farm (8°53′20″ N, 82°44′53″ W), located in the Renacimiento district, Chiriquí province, Panama, at altitudes ranging from 1590 to 1690 m above sea level, with average temperatures around 16 °C. Post-harvest, cherries were manually selected to remove defective fruits and then fermented at 22 °C for 96 h. The fermented cherries were dried under controlled conditions (24–30 °C, 50% relative humidity) for approximately two months. Dried beans were stored at 24 °C for one month before hulling using an Industrial Machinery TRC 46 kg machine (HNOS ARCE LTDA, Alajuela, Costa Rica), followed by classification and vacuum-packing for storage or roasting.

Roasting was carried out using a Roest sample roaster, 150 g capacity (ROEST, Oslo, Norway). The temperature ramp started at 150–180 °C and was maintained for approximately 7 min, reaching 193–205 °C, followed by a development phase of 55 s and subsequent cooling.

### 2.3. Preparation of the Extracts

The AErPGC was obtained following the Brazilian Pharmacopoeia extraction method [24], with slight modifications. Specifically, sample (4 g of dry material) was combined with 40 mL of distilled water and extracted using an Italian coffee maker. The resulting filtrate was frozen at −80 °C and subsequently lyophilized using a laboratory-scale freeze-dryer (Telstar LyoQuest, Telstar, Spain) under high vacuum until a constant weight was achieved, yielding a stable dry powder (AErPGC). The lyophilized extract was stored in a desiccator at room temperature, protected from light, until further use for phytochemical characterization and bioactivity assays.

### 2.4. Phytochemical Study

#### 2.4.1. Chemical Composition by HPLC MS/MS

Coffee sample analyses were performed on a Thermo Orbitrap QExactive Focus mass spectrometer coupled with Thermo Vanquish UHPLC. For HPLC analysis, the filtrates were reconstituted in an HPLC-grade solvent. A reversed-phase Ascentis express (C18) column—100 mm in length with a 2.1 mm internal diameter and a 2.7 μm particle size—was employed. The column was run at a flow rate of 0.4 mL/min and maintained at 30 °C. The mobile phase consisted of a gradient of water with 0.1% formic acid (A) and acetonitrile (B), with the following time and composition program: 0 min (95% A/5% B), 10 min (20% A/80% B), 15 min (0% A/100% B), 29 min (0% A/100% B), and 30 min (95% A/5% B). The total chromatographic run time was 40 min. For mass spectrometry detection, both positive and negative electrospray ionization modes were alternated, with mass ranging between 100 and 1000 amu, and a resolution of 70,000. In the positive mode, the electrospray voltage was 3.5 kV, and in the negative mode, 3.3 kV. To identify the major compounds, DDS fragmentation in Discovery mode was used, using a combination of three normalized collision energies of 10, 30, and 50, and a resolution for the fragments of 17,500.

Raw data processing, including peak detection, deconvolution, and alignment, was performed using MS-DIAL 4.9.221218 [25]. For the identification of chemical compounds, experimental MS/MS spectra were matched against the MassBank (Release 2024.06) [26]. For features requiring further structural elucidation, MS-FINDER [27] was utilized, incorporating in silico fragmentation rules and external database queries through ChemSpider [28].

#### 2.4.2. Total Polyphenols

For phenolic content analyses, an extract A was prepared by dissolving 50 mg of lyophilized AErPGC in 25 mL of distilled water. Total polyphenol content was determined using the Folin–Ciocalteu method, following Vega et al. (2017) [29]. An aliquot of 50 μL of Extract A was mixed with 3 mL of distilled water and 250 μL of Folin–Ciocalteu reagent (1 N). After 8 min of reaction, 750 μL of 20% Na_2_CO_3_ and 950 μL of distilled water were added. The mixture was incubated at room temperature for 30 min, and absorbance was measured at 765 nm using a UV–Vis spectrophotometer (PG Instruments Ltd., Wibtoft, UK, Model T70+). Quantification was performed using a gallic acid calibration curve, and results were expressed as milligrams of gallic acid equivalents per gram of lyophilized extract (mg GAE/g). All analyses were conducted in triplicate.

#### 2.4.3. Total Flavonoids

Flavonoid content was determined according to Vega et al. (2017) [29]. Briefly, 600 μL of Extract A was mixed with 2.58 mL of solution A (1.8 mL of 5% NaNO_2_ in 24 mL of water) and incubated for 5 min. Subsequently, 180 μL of 10% AlCl_3_ was added, and after 1 min, 2.52 mL of solution B (12 mL of 1 M NaOH in 14.4 mL of water) was added. Absorbance was measured immediately at 415 nm. Results were expressed as mg of catechin equivalents per gram of lyophilized extract (mg CE/g). All measurements were performed in triplicate.

#### 2.4.4. Tannin Content

Tannin content was determined using the AOAC 952.03 (Folin–Denis) method [30]. Briefly, 1 mL of Extract A was added to a 100 mL volumetric flask containing 75 mL of distilled water, followed by 5 mL of 1 N Folin–Ciocalteu reagent and 10 mL of saturated sodium carbonate solution. The volume was adjusted to 100 mL with distilled water, and the mixture was incubated at room temperature for 30 min. Absorbance was measured at 760 nm, and tannin content was calculated using a tannic acid calibration curve. Results were expressed as mg of tannic acid equivalents per gram of lyophilized extract (mg TAE/g). All measurements were performed in triplicate.

#### 2.4.5. Analysis of Polyphenols by HPLC–UV

Polyphenol analysis was performed by high-performance liquid chromatography (HPLC) using an Agilent 1260 Infinity system equipped with a quaternary pump, autosampler, and diode array detector (DAD), following the method of Çayan et al. (2020) [31] with minor modifications. Separation was achieved on a Zorbax SB-C8 column (150 × 4.6 mm, 5 µm) maintained at 40 °C. The mobile phases were: (A) water with 0.5% acetic acid, and (B) methanol with 0.5% acetic acid. The elution gradient was programmed as follows: 20% B (0 min), 50–58% B (1–2 min), 58–61% B (2–3 min), 61–63% B (3–5 min), 63–20% B (5–6 min), maintaining 20% B until 11 min. The flow rate was 1.5 mL min^−1^, the injection volume was 20 µL, and the detection wavelengths were 325 nm (caffeic acid), 275 nm (gallic acid), and 260 nm (vanillic and protocatechuic acids). Quantification was carried out using external calibration curves with pure standards. All analyses were conducted in triplicate.

#### 2.4.6. Analysis of Caffeine and Chlorogenic Acids by HPLC–UV

Caffeine and CGAs were analyzed using the same HPLC system, equipped with a Zorbax SB-C18 column (150 × 4.6 mm, 5 µm) maintained at 25 °C. The mobile phases were: (A) water with 0.02% trifluoroacetic acid, and (B) methanol with 0.02% trifluoroacetic acid. The elution gradient was programmed as follows: 8% B (0 min), 8–68% B (0–20 min), 68% B (20–23 min), followed by re-equilibration at 8% B for 15 min. The flow rate was 1.0 mL min^−1^, injection volume 20 µL of Extract A, and detection was performed at 325 nm (CGAs) and 280 nm (caffeine). Quantification was based on external calibration curves with analytical standards. All analyses were conducted in triplicate.

### 2.5. Antioxidant Activity

#### 2.5.1. DPPH Radical Scavenging Assay

The capacity of the extract to neutralize DPPH radicals was evaluated using a microplate-based assay [32]. The extract, at concentrations ranging from 0.48 to 1000 µg/mL, was mixed with a DPPH solution and incubated in the absence of light.

Absorbance was measured at 492 nm after 30 min. Quercetin was used as a reference antioxidant. Radical scavenging activity was expressed as percentage inhibition relative to a control. All assays were conducted in triplicate. The DPPH radical scavenging capacity was expressed as the percentage of inhibition, calculated using Formula (1).

(1) $$Free\;radical\;scavenging\left(\%\right)=\left[A_{control}-A_{test}\right]\times 100/A_{control}$$

where A_control_ is the absorbance of the reaction media without the test sample, and A_test_ is the absorbance in the presence of AErPGC or Quercetin.

#### 2.5.2. ABTS Radical Cation Scavenging Assay

The ABTS radical was generated by reacting ABTS with potassium persulfate, followed by incubation in darkness.

The AErPGC was incubated with the radical solution, and absorbance was recorded at 734 nm after 2 h. The assay was performed at different concentrations (from 0.48 to 1000 µg/mL), following the method previously described by Kinkela et al. (2026) [32]. Antioxidant activity was expressed as percentage inhibition, using quercetin as a positive control using Formula (1). All experiments were conducted in triplicate.

#### 2.5.3. NBT Superoxide Radical Scavenging Assay

The superoxide anion radical (O_2_^•−^) scavenging activity was evaluated using a non-enzymatic system, following the method described by Saha et al. [33]. The reaction mixture, containing the extract at varying concentrations (0.48 to 1000 µg/mL), was briefly incubated prior to measurement. Absorbance was recorded at 560 nm, and the percentage inhibition of superoxide generation was calculated relative to control conditions using the same formula applied for the DPPH radical inhibition assay. All experiments were performed in triplicate for each concentration tested.

#### 2.5.4. Lipid Peroxidation Inhibition Assay

The protective effect of AErPGC against lipid peroxidation was assessed using an egg yolk model [32,34]. The reaction system included egg yolk homogenate, ferrous ions, and the AErPGC at different concentrations, from 0.48 to 1000 µg/mL. Following incubation, the reaction was stopped with trichloroacetic acid, and malondialdehyde formation was quantified spectrophotometrically at 532 nm. Results were expressed as percentage inhibition of lipid peroxidation.

(2) $$Inhibition\;of\;lipoperoxidantion\left(\%\right)=\left[A_{control}-A_{test}\right]\times 100/A_{control}$$

where A_control_ is the absorbance of an egg yolk emulsion in a blank buffer without the test sample, and A_test_ is the absorbance of the egg yolk emulsion containing either the extracts or the standard substance (quercetin).

### 2.6. Vascular Bioactivity Screening of AErPGC

#### 2.6.1. Animal Conditions

All procedures involving animals were conducted in accordance with the Guide for the Care and Use of Laboratory Animals (NIH publication No. 85-23, revised 2011) and approved by the Comité de Ética de la Investigación y el Bienestar de los Animales de la Universidad de Panamá (CEIBA-UP-030-2022). Male Sprague Dawley rats (220–250 g) were used for the study. After euthanasia, the thoracic aorta was rapidly excised and immersed in ice-cold Krebs–Henseleit (K–H) buffer (in mM: NaCl 115.5, KCl 4.6, NaH_2_PO_4_ 1.3, NaHCO_3_ 24, CaCl_2_ 2.5, MgSO_4_ 1.2, glucose 11.1; pH 7.4), continuously aerated with 95% O_2_ and 5% CO_2_. Surrounding adipose and connective tissues were removed, and the aorta was cut into rings of 2–3 mm in length for vascular reactivity assays.

#### 2.6.2. Aortic Contraction Studies

Isolated aortic rings were mounted in a wire myograph system (Multi Wire Myograph System—620M) for the measurement of isometric tension. Vessels were maintained in K–H buffer at 37 ± 1 °C and continuously aerated with a gas mixture of 95% O_2_ and 5% CO_2_. After a 60 min equilibration period, rings were adjusted to their optimal resting tension (~9.81 mN). Endothelial integrity was verified by acetylcholine (ACh, 10^−4^ M)-induced relaxation in tissues pre-contracted with phenylephrine (PE, 10^−6^ M); preparations showing ≥70% relaxation were considered endothelium-intact. In selected experiments, the endothelium was mechanically removed by gently rubbing the luminal surface with fine forceps.

Vascular responses were evaluated by precontracting aortic rings with PE (10^−6^ M) or KCl (80 mM), followed by cumulative addition of AErPGC (100–3000 μg/mL) or vehicle (K–H buffer). Changes in vascular tension were continuously recorded using a PowerLab/400 data acquisition system (ADInstruments, Colorado Springs, CO, USA).

### 2.7. Toxicity Assays

#### 2.7.1. Preliminary Toxicity Assessment Using the *Artemia salina* Leach Bioassay

Preliminary toxicity was assessed using *Artemia salina* nauplii, following the methodology previously described by Kinkela et al. (2026) [32]. Larvae were exposed to different concentrations of AErPGC (0.48 to 1000 µg/mL), and mortality was recorded after 24 h.

The median lethal dose (LD_50_) was defined as the concentration capable of inducing 50% mortality in the nauplii.

#### 2.7.2. Single Dose Acute Oral Toxicity Study in Rat

The acute toxicity of AErPGC was evaluated according to OECD Test Guideline 423 [35]. Although the guideline recommends starting at 300 mg/kg in the absence of prior data, a single oral dose of 2 g/kg was selected based on previous studies demonstrating the safety of coffee extracts at this concentration [36]. Male and female rats were divided into treatment groups (AErPGC 2 g/kg) and control groups (distilled water). Treatments were administered orally by using gavage in a single dose.

Animals were monitored over a 14-day period for clinical signs of toxicity and mortality. At the end of the study, major organs were collected for macroscopic evaluation and relative weight determination.

### 2.8. Statistical Analysis

Data are presented as mean ± standard deviation (SD). Statistical analyses were performed using one-way or two-way ANOVA, followed by Student’s *t*-test (two-tailed) for pairwise comparisons when appropriate. Antioxidant and toxicity assays were conducted in triplicate. For vascular screening, *n* refers to the number of vessels from different animals. Differences were considered statistically significant at *p* < 0.05. All analyses were carried out using GraphPad Prism 10.0.

## 3. Results

In this study, AErPGC was chemically characterized, and the antioxidant and vascular activity were additionally investigated to determine the nutraceutical efficacy in the field of vascular therapy.

### 3.1. Chemical Composition of AErPGC by HPLC MS/MS

Chromatographic and mass spectrometric analyses led to the identification of 57 compounds in the AErPGC sample, categorized into 12 major (more than 1.30%), 2 minor (between 0.9 and 0.1%), and 17 trace (<0.1%) constituents. Table 1 summarizes the total composition (expressed as percentages of the area), representing the average of three replicates.

The phytochemical analysis of AErPGC revealed that caffeine was the most abundant compound, accounting for 37.40% of the total composition, followed by trigonelline (14.25%), a characteristic alkaloid commonly associated with lightly roasted coffee beans. Additionally, (+)-quinic acid was present at a relatively high proportion (9.61%), suggesting partial degradation of chlorogenic acids during roasting.

Chlorogenic acids (CGAs) and related derivatives were also identified, comprising a diverse group of caffeoyl- and feruloyl-based compounds. Among these, 5-O-caffeoylquinic acid was the predominant caffeoylquinic acid (3.80%), followed by 3-*O*-caffeoylquinic acid (1.32%) and 4-*O*-caffeoylquinic acid (1.38%). Feruloylquinic acid derivatives were also significant, including 3-*O*-feruloylquinic acid (3.48%), 5-*O*-feruloylquinic acid (1.30%), and an additional feruloylquinic acid isomer (1.30%). Minor amounts of dicaffeoyl derivatives, such as 3,5-*O*-dicaffeoylquinic acid (0.09%) and 3,4-*O*-dicaffeoyl-1,5-quinolactone (0.35%), were also detected.

Furthermore, caffeoylshikimic acid derivatives, including 4-*O*-caffeoylshikimic acid (3.79%) and 5-*O*-caffeoylshikimic acid (3.80%), were identified, along with 4-coumaroylshikimic acid (0.07%).

**Figure 1:**
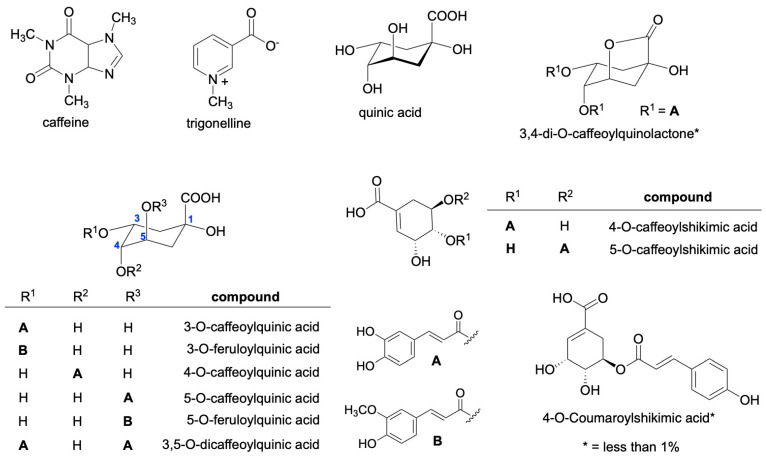
Structures of the most representative compounds in AErPGC.

Figure 1 illustrates the chemical structures of compounds present at concentrations exceeding 1%. Additionally, considering the notable antioxidant potential of CGAs, the structures of some compounds identified at levels below 1% are also included for comprehensive reference.

### 3.2. Total Phenolic Content

The total phenolic content on AErPGC is shown in Table 2.

### 3.3. Chlorogenic Acids, Caffeine, and Phenolic Compounds Content by HPLC-UV

The results of the quantification of phenolic acids and alkaloids present in the AErPGC are summarized in Table 3. Isomers like caffeoylquinic acids (CQA) and feruloylquinic acids (FQA) the identification was based on a combination of exact mass, retention time (RT), and characteristic MS/MS fragmentation patterns.

### 3.4. Antioxidant Activity

The antioxidant capacity of AErPGC was assessed using multiple radical scavenging assays (DPPH, ABTS, and superoxide), with quercetin as a reference compound. Results are summarized in Table 4 as maximal inhibitory efficacy (E_max_) and IC_50_ values.

**Figure 2:**
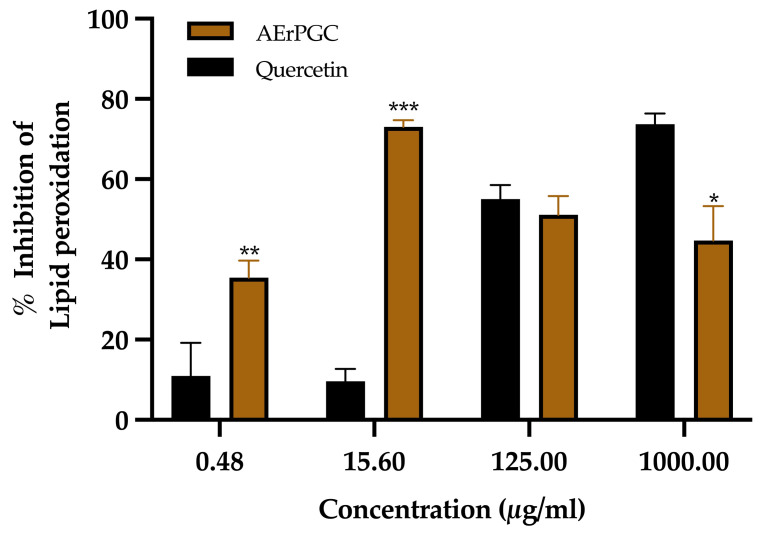
Effects of AErPGC on lipid peroxidation assay. Data are presented as the mean ± SD for an *n* = 3. * *p*  ≤  0.05; ** *p*  ≤  0.01; *** *p*  ≤  0.001 vs. Quercetin.

The highest antioxidant activity was observed against the ABTS radical. At the highest concentration tested, AErPGC exhibited a scavenging effect statistically comparable to that of quercetin; however, its overall potency was lower, as indicated by a higher EC_50_ value (182 ± 4.1 µg/mL for AErPGC vs. 15.5 ± 8.6 µg/mL for quercetin). Although inferior to the standard quercetin, the extract demonstrated a moderate ability to neutralize the superoxide anion (82.3 ± 1.4 vs. 62.4 ± 2.9, respectively). In the lipid peroxidation inhibition assay, quercetin exhibited a clear concentration-dependent inhibitory effect. In contrast, AErPGC showed strong antioxidant activity at low concentrations, inhibiting lipid peroxidation by 72.71 ± 1.63% at 15.6 µg/mL, with a gradual reduction in inhibitory intensity observed at higher concentrations (Figure 2). This pattern nevertheless reflects a higher inhibitory potency and overall efficacy of AErPGC compared with the standard compound.

### 3.5. Vascular Bioactivity Screening of AErGC

The vascular effects of AErPGC were evaluated by assessing its relaxant responses against phenylephrine (PE) and potassium chloride (KCl)-induced contractions in isolated rat aortic rings, with and without endothelium. Maximal effect (E_max_) and potency (EC_50_) values were calculated to quantify the degree of vasorelaxation (Table 5).

**Figure 3:**
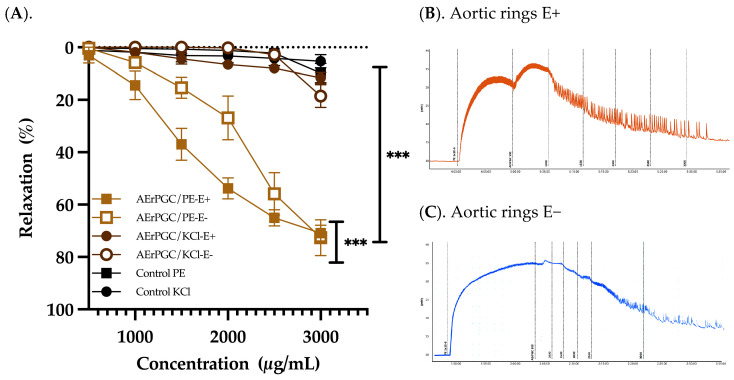
Concentration-dependent relaxant effects of AErPGC (**A**) on phenylephrine (PE, 1 × 10^−6^ M) or KCl (80 mM) precontracted rat aortic rings. Representative traces showing vascular response induced by cumulative concentrations of AErPGC in aortic rings with endothelium (**B**) and without endothelium (**C**) precontracted with PE. Values are expressed as mean ± SD (*n* = 6). *** p < 0.001 versus control PE.

In PE-precontracted rings, AErPGC elicited significant endothelium-dependent vasorelaxant activity, with an E_max_ of 70.84% ± 2.9 and an EC_50_ of 1940 µg/mL (Table 5, Figure 3A). A comparable maximal response was also observed in endothelium-denuded preparations (E_max_ = 72.62% ± 6.8, EC_50_ = 2402 µg/mL), suggesting both endothelium-dependent and -independent mechanisms.

In contrast, in KCl- precontracted rings, the maximal vasorelaxant effect of AErPGC was substantially attenuated, reaching 11.59 ± 1.9% in endothelium-intact rings and 18.52 ± 8.4% in endothelium-denuded rings (Table 5, Figure 3A). These results indicate that membrane depolarization markedly limits the vasorelaxant response to AErPGC.

### 3.6. Toxicity Assays

#### 3.6.1. Lethality Effects of AErPGC on *Artemia salina*

No lethality was observed in *Artemia salina* nauplii at concentrations up to 1000 µg/mL. Based on Meyer’s toxicity criteria [37], AErPGC can be classified as non-toxic, and further determination of LC_50_ values was not required.

#### 3.6.2. Single Dose Acute Oral Toxicity Study

**Figure 4:**
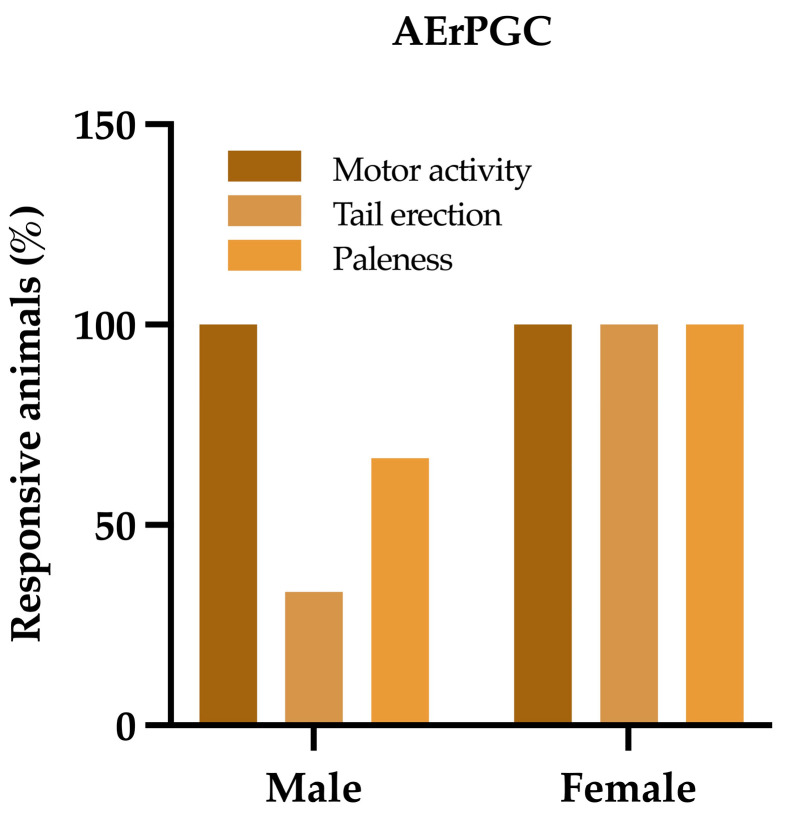
Main clinical effects observed during acute toxicity study on Sprague-Dawley rats exposed to AErPGC by gavage.

Following oral administration of AErPGC, transient clinical signs -including increased motor activity, tail erection, and pallor- were observed within the first 15 min (Figure 4). These effects resolved spontaneously within the first hour. No mortality or persistent signs of toxicity were recorded during the 14-day observation period. Additionally, no macroscopic alterations were detected in major organs, and organ weight ratios remained unchanged (Table 6). Collectively, these findings indicate that AErPGC does not induce acute toxicity under the experimental conditions evaluated.

## 4. Discussion

Coffee remains one of the most widely consumed beverages worldwide [38]. In June 2025, global green coffee bean exports reached 10.23 million bags, representing a 3.3% increase compared with June 2024 [39]. The two main coffee species are *Coffea arabica* and *Coffea canephora* (Robusta) [40], with *C. arabica* being particularly valued for its superior sensory quality and higher content of bioactive compounds. One of the main compositional differences between these species is lipid content, which is typically higher in Arabica (approximately 15%) than in Robusta (around 10%) [41]. Coffee lipids, together with other constituents, contribute substantially to the formation of aromatic compounds during roasting, shaping the sensory profile of the beverage [42]. Among Arabica varieties, Panamanian Geisha coffee occupies a prominent position in the specialty coffee market due to its exceptional sensory attributes and high economic value [19].

In this context, phytochemical characterization of AErPGC revealed the presence of furanic compounds such as methyl 2,5-di-*O*-acetyl-3,6-dideoxyhexofuranoside, which are known to contribute caramel-like notes characteristic of roasted coffee. It is important to emphasize that variations in roasting profiles can markedly influence the formation and degradation of aromatic compounds, leading to distinct sensory outcomes. Given that aroma is one of the defining and most desirable attributes of high-quality Panamanian Geisha coffee [19], the chemical characterization presented herein provides objective support for the distinctive sensory properties traditionally attributed to this specialty coffee.

Beyond organoleptic constituents, this study also focused on compounds associated with the nutraceutical potential of coffee, particularly caffeine and CGAs [43]. The evaluation of vasodilatory and antioxidant properties of Panamanian Geisha coffee extract is supported by a substantial body of evidence linking coffee consumption with beneficial cardiovascular effects [44]. In this regard, Zhou et al. (2025) demonstrated that moderate coffee intake (1–3 cups per day) is associated with a significant reduction in both all-cause and cardiovascular mortality over a five-year period, particularly among individuals with pre-existing hypertension, cardiovascular disease, or cerebrovascular events [45]. These findings reinforce the concept of coffee as a promising candidate for inclusion in the diet as a functional food [46] and underscore the importance of elucidating the mechanisms underlying its cardioprotective effects.

CGAs—including 3-*O*-caffeoylquinic acid (3-CQA), 4-*O*-caffeoylquinic acid (4-CQA), 5-*O*-caffeoylquinic acid (5-CQA), 4-*O*-feruloylquinic acid (4-FQA), 5-*O*-feruloylquinic acid (5-FQA), 3,5-*O*-dicaffeoylquinic acid (3,5-DiCQA), and 4,5-*O*-dicaffeoylquinic acid (4,5-DiCQA)— constitute the predominant class of polyphenols in coffee and are well known for their potent antioxidant activity and cardioprotective effects [47]. Previous studies have reported the presence of phenolic compounds in both beans and leaves of Geisha coffee, with higher concentrations typically found in the leaves [21]. In AErPGC, 5-CQA was identified as the predominant CGA, in agreement with observations reported by Jeon et al. (2024) for this coffee variety [21]. Notably, significant levels of 3-CQA and 4-CQA were also detected. This finding is particularly relevant given that CGAs are known to undergo degradation during roasting, with their concentration generally decreasing as roasting intensity increases [48]. These processes may alter the physicochemical behavior of the beans during roasting and influence the preservation or transformation of bioactive compounds. Consistent with this interpretation, previous studies have suggested that natural coffee aromatization and fermentation techniques can modulate the levels of bioactive constituents, potentially enhancing the health-promoting properties of coffee [49,50,51].

The results of the present study demonstrate that AErPGC exhibits a pronounced antioxidant activity, albeit with differential efficacy against distinct free radicals. Particularly noteworthy is the antiradical activity against the ABTS radical, where AErPGC achieved an efficacy comparable to that of the quercetin standard. Even more striking was its ability to inhibit lipid peroxidation, as AErPGC not only showed the highest inhibitory effect but also surpassed quercetin in potency. These findings are consistent with the known chemical composition of Panamanian Geisha coffee, whose antioxidant properties have been attributed to the combined action of caffeine, CGAs, and other constituents such as trigonelline and the diterpene kahweol [52,53,54,55]. Additionally, the antioxidant activity reported for compounds such as caffeic acid [56], ferulic acid [57,58], and kahweol [55,59] may act synergistically, contributing to the marked efficacy of AErPGC in lipid peroxidation inhibition.

Given the growing interest in *Coffea arabica* as a source of bioactive compounds with therapeutic potential, previous studies have also documented significant antioxidant effects for Arabica coffee extracts [60]. In this context, investigations comparing different Arabica varieties have shown that Geisha coffee exhibits ABTS scavenging activity comparable to that observed for AErPGC [21]. Direct comparisons with other studies, however, remain challenging due to differences in experimental objectives and methodologies. While some studies focus on regional variations in coffee antioxidant profiles [61], others assess the impact of extraction techniques [62] or compare antioxidant activity among different plant tissues, such as leaves and green fruits [21].

In parallel with chemical characterization, this study sought to evaluate the cardiovascular relevance of the bioactive compounds identified in roasted Panamanian Geisha coffee extract. The investigation of vasodilatory and antioxidant effects is supported by growing evidence that coffee consumption exerts protective actions on the cardiovascular system [63]. Such evidence has further strengthened the proposal of coffee as a functional food with potential cardiovascular benefits [64]. 

Importantly, the present study provides new evidence demonstrating that AErPGC exerts a vasodilatory effect through both endothelium-dependent and endothelium-independent mechanisms. These findings are consistent with previously reported effects of caffeine, a major constituent of AErPGC, which has been shown to enhance endothelium-dependent vasodilation in isolated aortic rings [65] and in healthy young men [66]. Conversely, endothelium removal has been demonstrated to attenuate caffeine-induced vasorelaxation in vascular preparations [65], underscoring the contribution of endothelial mechanisms to its vasodilatory effects.

Other bioactive constituents identified in AErPGC, including CGAs and caffeic acid, may also contribute to and potentiate the observed vasodilatory response. Caffeic acid has been reported to induce vasodilation exceeding 90% in isolated aortic rings [67], while CGAs have been shown to promote endothelium-dependent vasodilation mediated by nitric oxide synthase (NOS) and cyclooxygenase (COX) signaling pathways [68]. Furthermore, dietary supplementation with CGAs has been demonstrated to attenuate endothelial dysfunction and hypertension in spontaneously hypertensive rats, effects that were associated with reduced oxidative stress and improved nitric oxide bioavailability [69]. Collectively, these bioactive compounds have been implicated in the antihypertensive effects [70,71] and the preservation of endothelial function [72,73] observed following coffee extract administration.

In addition to the biological activities described the safety profile of AErPGC represents a key aspect for its potential application. The absence of lethality in the Artemia salina bioassay at concentrations up to 1000 µg/mL suggests a low level of general toxicity, which is consistent with the acute oral toxicity results showing no mortality or persistent adverse effects. Additionally, the lack of macroscopic organ alterations and stable organ weight ratios indicates no evident systemic toxicity under the tested conditions. These findings support a low acute toxicity profile and highlight the potential of AErPGC for nutraceutical applications, although further studies are required to confirm its long-term safety.

## 5. Conclusions

This study provides the first comprehensive characterization of roasted Panamanian Geisha coffee, establishing a robust efficacy profile against biologically relevant radicals, such as O_2_•^−^ and lipid peroxidation. Our findings demonstrate that AErPGC possesses potent antioxidant and vasorelaxant activities, supporting the antihypertensive potential of this specialty cultivar. These findings broaden current knowledge and highlight new opportunities for further investigation into the cardiovascular effects of Geisha specialty coffee. Ultimately, the pronounced bioactivity and safety profile of Panamanian Geisha coffee also underscores the need to elucidate the underlying mechanisms responsible for these activities and emphasizes the potential translational relevance of these results for the development of nutraceutical preparations derived from Panamanian Geisha coffee.

## Figures and Tables

**Table 1:** Phytochemical constituents of the AErPGC determined by HPLC-MS.

Nº	Name	Formula	MW	RT	Area (%)	[M + H]^+^	[M − H]^−^
1	Valinol	C_5_H_13_NO	103.0996	0.51	0.98	104.1071	
2	Sucrose	C_12_H_22_O_11_	342.1116	0.52	0.61		341.1088
3	(+)-Quinic acid	C_7_H_12_O_6_	192.0632	0.54	9.61		191.0554
4	Trigonelline	C_7_H_7_NO_2_	137.0470	0.55	14.25	138.0548	
5	Unknown	C_9_H_6_O_3_	162.0309	0.61	5.50	163.0387	
6	3-*O*-Caffeoylquinic acid	C_16_H_18_O_9_	354.0943	0.64	1.32	355.1021	353.0876
7	Caffeine	C_8_H_10_N_4_O_2_	194.0796	0.68	37.40	195.0874	
8	5-*O*-Caffeoylshikimic acid	C_16_H_16_O_8_	336.0851	1.74	3.80		335.0874
9	3-*O*-Feruloylquinic acid	C_17_H_20_O_9_	368.1110	1.85	3.48		367.1032
10	5-*O*-Caffeoylquinic acid	C_16_H_18_O_9_	354.0943	1.92	3.80	355.1021	353.1027
11	Cyclo(leucylprolyl)	C_11_H_18_N_2_O_2_	210.1362	2.09	0.20	211.1332	
12	4-*O*-Caffeoylquinic acid	C_16_H_18_O_9_	354.0943	2.27	1.38	355.1021	353.0971
13	4-*O*-Caffeoylshikimic acid	C_16_H_16_O_8_	336.0849	2.28	3.79		335.0771
14	5-*O*-Feruloylquinic acid	C_17_H_20_O_9_	368.1110	2.39	1.30		367.1032
15	Cyclo(isoleucylprolyl)	C_11_H_18_N_2_O_2_	210.1361	2.40	0.25	211.1439	
16	Aconine	C_25_H_41_NO_9_	499.2772	2.47	0.21	500.2850	
17	Atractyloside II	C_25_H_38_O_9_	482.2520	2.48	0.46		481.2442
18	Unknown	C_18_H_33_N_3_O_3_	339.2516	2.58	0.19	340.2594	
19	Feruloylquinic acid isomer	C_17_H_20_O_9_	368.1113	2.75	1.30		367.1035
20	Ferulic acid	C_10_H_10_O_4_	194.0580	2.80	0.28		193.0502
21	(3S,5S,7R,9R,11S,13S)-2,2,6,6,10,10,14,14-Octamethyl-1,3,5,7,9,11,13,15-pentadecaneoctol	C_23_H_48_O_8_	452.3356	3.12	0.10	453.3434	
22	Unknown	C_20_H_24_O_4_	328.1667	3.18	0.14	329.1745	
23	Nymphaeoside A	C_27_H_38_O_12_	554.2369	3.20	0.08		553.2291
24	4-*O*-Coumaroylshikimic acid	C_16_H_16_O_7_	320.0901	3.22	0.07		319.0823
25	Unknown	C_17_H_18_O_8_	350.1007	3.36	0.26		349.0929
26	Unknown	C_12_H_12_O_6_	252.0640	3.41	0.10		251.0562
27	3,4-*O*-Dicaffeoyl-1,5-quinolactone	C_25_H_22_O_11_	498.1155	3.42	0.35	499.1233	497.1092
28	Unknown	C_12_H_12_O_3_	204.0782	3.45	0.23	205.0860	203.0710
29	3,5-*O*-Dicaffeoylquinic acid	C_25_H_24_O_12_	516.1261	3.92	0.09	517.1339	515.1194
30	Unknown	C_10_H_8_O_4_	192.0419	4.07	0.05	193.0497	
31	Cnidimoside A	C_21_H_26_O_10_	438.1531	4.15	0.09		437.1453
32	Unknown	C_27_H_36_O_11_	536.2264	4.20	0.06		535.2186
33	5-Geranyloxy-7-methoxycoumarin	C_20_H_24_O_4_	328.1667	4.21	0.11	329.1745	
34	Atractyloside I	C_36_H_56_O_15_	728.3625	4.39	0.58		727.3547
35	Methyl 2,5-di-*O*-acetyl-3,6-dideoxyhexofuranoside	C_11_H_18_O_6_	246.1098	4.42	0.19	247.1176	
36	N-Caffeoyltryptophan	C_20_H_18_N_2_O_5_	366.1210	4.49	0.09	367.1288	365.1143
37	Kahweol	C_20_H_26_O_3_	314.1876	4.51	0.05	315.1954	
38	Unknown	C_14_H_31_NO_2_	245.2349	4.66	0.06	246.2427	
39	Unknown	C_16_H_35_NO_3_	289.2612	4.77	0.03	290.2690	
40	Unknown	C_11_H_10_O_4_	206.0574	4.80	0.10	207.0652	
41	*N-p*-Hydroxycoumaroyltryptophan	C_20_H_18_N_2_O_4_	350.1261	4.94	0.02	351.1340	
42	Atractyloside III	C_30_H_46_O_10_	566.3096	5.01	0.15		565.3018
43	Unknown	C_20_H_30_O_4_	334.2152	5.12	0.03		333.2074
44	Unknown	C_16_H_35_NO_2_	273.2660	5.66	0.87	274.2738	
45	Unknown	C_15_H_22_O	218.1664	5.69	0.03	219.1742	
46	Unknown	C_14_H_31_NO	229.2400	5.71	0.09	230.2478	
47	Phytosphingosine	C_18_H_39_NO_3_	317.2922	5.75	0.43	318.3000	
48	Unknown	C_20_H_43_NO_4_	361.3186	5.85	0.07	362.3264	
49	Unknown	C_18_H_39_NO_2_	301.2975	6.58	0.12	302.3053	
50	2-Aminoicosane-1,3,4-triol	C_20_H_43_NO_3_	345.3237	6.63	0.05	346.3315	
51	Unknown	C_23_H_47_NO_4_	401.3497	6.67	1.62	402.3575	
52	Unknown	C_17_H_26_O_4_	294.1838	6.71	0.07		293.1760
53	Unknown	C_23_H_48_N_2_O	368.3758	8.04	1.35	369.3836	
54	Unknown	C_23_H_48_N_2_O_2_	384.3708	8.36	0.61	385.3786	
55	Unknown	C_28_H_53_NO_7_	515.3812	8.75	0.34	516.3890	
56	Unknown	C_30_H_51_N_5_O_5_	561.3880	8.76	0.59	560.3802	
57	Unknown	C_35_H_63_NO_3_	545.4801	10.31	0.41	546.4879	

**Table 2:** Total polyphenol, flavonoid, and tannin contents in lyophilized AErPGC.

Sample	Total Polyphenols (mg GAE/g Lyophilized Extract)	Flavonoids (mg CE/g Lyophilized Extract)	Tannins (mg TAE/g Lyophilized Extract)
AErPGC	170.1 ± 1.3	171.9 ± 3.6	161.5 ± 3.7

GAE: gallic acid equivalents; CE: catechin equivalents; TAE: tannic acid equivalents. Results are expressed as mean ± standard deviation (*n* = 3).

**Table 3:** Concentrations (mg/g lyophilized extract) of chlorogenic acids, caffeine, and phenolic of phenolic acids and alkaloids from AErPGC.

Sample	3-CQA	5-CQA	4-CQA	Caffeine	Gallic Acid	Caffeic Acid	Vanillic Acid	Protocatechuic Acid
AErPGC	35.8 ± 2.9	74.5 ± 6.9	38.5 ± 3.4	69.5 ± 6.4	0.8 ± 0.01	5.6 ± 0.2	<DL	<DL

CQA: caffeoylquinic acids. Detection limits (DL): gallic acid = 9.97 × 10^−5^ mg/mL; caffeic acid = 1.31 × 10^−4^ mg/mL; vanillic acid = 2.98 × 10^−4^ mg/mL; protocatechuic acid = 4.64 × 10^−5^ mg/mL. Results are expressed as mean ± standard deviation (*n* = 3).

**Table 4:** E_max_ and IC_50_ values compared to different antioxidant assays of AErPGC and Quercetin.

	DPPH		ABTS		O_2_^•−^	
	E_max_ (%)	IC_50_ (μg/mL)	E_max_ (%)	IC_50_ (μg/mL)	E_max_ (%)	IC_50_ (μg/mL)
AErPGC	25.1 ± 6.1 *	14.7 ± 4.9 *	86.3 ± 0.9	182.9 ± 4.1 *	62.4 ± 2.9 *	76.91 ± 9.4 *
Quercetin	80.4 ± 1.2	44.1 ± 7.3	86.9 ± 0.1	15.5 ± 8.6	82.3 ± 1.4	22.72 ± 6.6

Data are presented as mean ± SD for *n* = 3. * *p* < 0.05 vs. Quercetin.

**Table 5:** E_max_ and IC_50_ value of AErPGC on PE and KCl-precontracted endothelium-intact and endothelium-denuded aortic rings.

	PE				KCl			
	With Endothelium		Without Endothelium		With Endothelium		Without Endothelium	
Extract	E_max_ (%)	IC_50_ (μg/mL)	E_max_ (%)	IC_50_ (μg/mL)	E_max_ (%)	IC_50_ (μg/mL)	E_max_ (%)	IC_50_ (μg/mL)
AErPGC	70.84 ± 2.9 ***	1940	72.62 ± 6.8 ***	2402	11.59 ± 1.9	Nd	18.52 ± 8.4	Nd
Control-K-H	9.82 ± 8.8	Nd	7.37 ± 8.1	Nd	5.31 ± 4.9	Nd	5.37 ± 6.8	Nd

Values are expressed as mean ± SD of six aortic rings (*n* = 6) in each group. *** *p* < 0.001 compared to control (K-H). Nd = not determined.

**Table 6:** Effects of AErPGC on relative weights of organs [(organ weight/body weight) × 100%] after 14 days.

Grupo		Heart	Liver	Lungs	Left Kidney	Spleen
Male	Control	0.36 ± 0.04	3.75 ± 0.26	0.41 ± 0.01	0.81 ± 0.05	0.24 ± 0.03
	AErPGC	0.35 ± 0.01	3.98 ± 0.21	0.40 ± 0.02	0.73 ± 0.03	0.22 ± 0.02
Female	Control	0.38 ± 0.06	3.90 ± 0.49	0.49 ± 0.08	0.83 ± 0.09	0.56 ± 0.07
	AErPGC	0.38 ± 0.01	4.11 ± 0.17	0.52 ± 0.04	0.90 ± 0.04	0.66 ± 0.05

Values are presented as mean  ±  SEM of triplicates (*n*  =  3).

## Data Availability

The original contributions presented in the study are included in the article, further inquiries can be directed to the corresponding authors.

## Abbreviations

The following abbreviations are used in this manuscript:

AErPGC	Aqueous Extract of Roasted Panamanian Geisha Coffee
ABTS	2,2′-Azino-bis(3-ethylbenzothiazoline-6-sulfonic acid)
ACh	Acetylcholine chloride
DMSO	Dimethyl sulfoxide
DMEM	Dulbecco’s Modified Eagle Medium
DPPH	2,2-Diphenyl-1-picrylhydrazyl
IC_50_	Half-maximal Inhibitory Concentration
K–H buffer	Krebs–Henseleit buffer
LD_50_	Median Lethal Dose
L-NAME	NG-nitro-L-arginine methyl ester
MDA	Malondialdehyde
MTT	3-(4,5-dimethylthiazol-2-yl)-2,5-diphenyltetrazolium bromide
NADH	Nicotinamide adenine dinucleotide
NBT	Nitroblue tetrazolium
OECD	Organisation for Economic Co-operation and Development
PE	Phenylephrine hydrochloride
PMS	Phenazine methosulfate
SNP	Sodium nitroprusside
Trolox	6-Hydroxy-2,5,7,8-tetramethylchroman-2-carboxylic acid
